# Lactobacillus rhamnosus Bacteremia in an Immunocompromised Renal Transplant Patient

**DOI:** 10.7759/cureus.6887

**Published:** 2020-02-05

**Authors:** Selin Sendil, Isha Shrimanker, Qurat Mansoora, John Goldman, Vinod K Nookala

**Affiliations:** 1 Internal Medicine, University of Pittsburgh Medical Center Pinnacle, Harrisburg, USA; 2 Internal Medicine: Infectious Disease, University of Pittsburgh Medical Center Pinnacle, Harrisburg, USA

**Keywords:** bacteremia, lactobacillus rhamnosus, renal transplant

## Abstract

Conventionally, Lactobacillus species are considered as low virulence organisms and rarely cause infection in immunocompetent individuals. However, it might be an opportunistic infection source in immunocompromised patients and can cause invasive serious infections. To our knowledge, there are only a handful of cases in the literature reporting primary bloodstream infection caused by Lactobacilli spp. in transplant recipients. Here, we report a case of a kidney transplant recipient with Lactobacillus rhamnosus bacteremia.

## Introduction

Lactobacillus spp. are facultatively anaerobic, gram-positive non-spore-forming rods that are a part of the normal human commensal microbiota of the oral, gastrointestinal, and female genital tract [1]. Some Lactobacillus spp. are used in probiotic products for potential benefits related to fermentative properties with limited scientific evidence to support their use [2].

Conventionally, Lactobacillus spp. are deemed to be of low virulence; however, a number of publications point out potential harmful effects with their use ranging from gastrointestinal side effects to invasive systemic infections such as peritonitis, deep abdominal abscesses, and bacteremia [1,3,4]. In some reports from Europe, Lactobacillus rhamnosus (L. rhamnosus) was the most common species involved in Lactobacillus bacteremia [4]. We report a case of L. rhamnosus bacteremia in an elderly immunosuppressed male with multiple comorbidities.

## Case presentation

A 75-year-old male with end-stage renal disease secondary to type 2 diabetes mellitus who received deceased donor renal transplant in 2012 presented with worsening kidney function, altered mental status, severe septic shock, and respiratory failure. The patient was under maintenance immunosuppression with tacrolimus 3.5 mg twice daily, mycophenolate mofetil 180 mg twice daily, and prednisone 5 mg once daily. Past medical history was also significant for stroke (status post carotid angioplasty), coronary artery disease, and essential hypertension. He was transferred to the intensive care unit (ICU) from a long-term acute care facility (LTACH). At the LTACH facility, he was treated with oral vancomycin for Clostridium difficile (C. difficile) infection, along with intravenous vancomycin and meropenem for right heel osteomyelitis. Six months before the ICU admission, he was found to have acute tubular necrosis, proven by kidney biopsy, and receiving intermittent hemodialysis.

On arrival to the ICU, his pulse was 118 beats per minute, blood pressure 119/69 mmHg, respiratory rate of 12 breaths per minute, and oxygen saturation of 100% on room air. On physical exam, he had bilateral pedal edema. Laboratory investigations revealed hemoglobin of 7.9 g/dl, potassium of 7.1, blood urea nitrogen 60 mg/dl, and creatinine 1.91 mg/dl. Urine analysis revealed leukocyte esterase +1, red blood cells 16-30/hpf, white blood cells 6-10/hpf, and urine bacteria +1. The patient was in severe septic shock requiring five vasopressors and initially treated with broad-spectrum antibiotics (intravenous vancomycin, cefepime, and metronidazole). Both blood culture bottles grew L. rhamnosus, and anti-biotherapy was narrowed to ampicillin alone.

The identification of L. rhamnosus was performed by the 16S rDNA sequence analysis by the collaborating laboratory. CT of the abdomen and pelvis was negative for a source of bacteremia. Repeat blood cultures grew yeast. Anti-biotherapy broadened to micafungin, vancomycin, and piperacillin/tazobactam. While he was in the LTACH facility, his kidney function progressively worsened, and he required emergent hemodialysis. During his ICU stay, he was on continuous renal replacement therapy intermittently. His overall clinical status improved, and the vasopressors were weaned off. He was successfully extubated. However, he remained encephalopathic. His family decided to continue with comfort measures only and he passed away in a few days.

## Discussion

Lactobacillus spp. has been considered an opportunistic pathogen in immunocompromised patients, and it has the propensity to cause invasive infections such as bacteremia, abscess, and endocarditis. Other reported risk factors include intravenous catheters, prior hospitalization or surgery, and broad-spectrum antibiotic use [4]. In the study by Salminen et al., overall mortality was found to be 26% at one month and 48% at one year [4]. Naqvi et al. reported a case of fatal L. rhamnosus endocarditis involving a young patient with a history of complicated cirrhosis and prior C. difficile colitis [5]. The patients with loss of integrity of the intestinal mucosal barrier are also at increased risk; hence, several occurrences of Lactobacillus bacteremia have been reported in patients with ulcerative colitis [6]. Gut translocation and systemic dissemination of organisms may be the underlying pathogenesis for invasive infections in immunocompromised patients [7]. L. rhamnosus strains have been found to induce platelet aggregations, have a modified exopolysaccharide cluster, and form strong biofilms [8,9].

Lactobacillus bacteremia has been reported in patients with acute myeloid leukemia, large granular lymphocytic leukemia, and in transplant recipients [10]. Recurrent Lactobacillus spp. bacteremia with chronic lymphocytic leukemia has been reported. The Lactobacilli isolated from blood cultures were susceptible to penicillin and gentamicin, and the patient was treated with this regimen. The authors concluded that despite intermittent fever-free periods and negative blood cultures after treatment, successful eradication of the underlying source is doubtful given his recurrent bacteremia. Therefore, the patient was ultimately placed on life-long oral amoxicillin-clavulanate therapy for prophylaxis [11].

Our patient received a renal transplant in 2013 and was under maintenance immunosuppression regimen with tacrolimus, mycophenolate mofetil, and prednisone. The review of our patient’s chart revealed that he had been taking probiotic supplements containing Lactobacillus acidophilus and Lactobacillus bulgaris for five months before his transfer to the ICU. Probiotic pills were not sent for testing due to unavailability; however, colonization by probiotic use long after consumption stopped is highly questionable. Also, these strains are different from those detected in blood cultures. To our knowledge, there are only two more case reports in the literature reporting primary bloodstream infection caused by Lactobacilli spp. in a kidney transplant recipient [12,13]. Vanichanan et al. reported a 60-year-old renal transplant recipient who developed an intra-abdominal abscess which grew a carbapenem-resistant Lactobacillus casei (L. casei), with an uneventful follow-up course for four years. He had been taking an over-the-counter probiotic for six months, until two months before presentation when he developed abdominal discomfort due to enlarging native kidneys. He underwent bilateral native nephrectomy and was discharged home. One week later, he returned to the hospital in septic shock and respiratory failure. The culture of the perihepatic fluid grew L. casei. In addition, the probiotic supplement taken by the patient also grew a similar organism raising the concern of probiotic-associated infection in immunocompromised individual [13].

The treatment of invasive, severe, or recurrent lactobacillus infections can be challenging. Many strains of Lactobacillus, including L. rhamnosus, are intrinsically resistant to vancomycin. Resistance to ciprofloxacin, tetracycline, meropenem, metronidazole, and sulfonamides has been reported, with some isolates exhibiting intermediate resistance to linezolid [14].

In recent years, the use of probiotics has increased worldwide for the treatment of infantile and adult diarrhea, C. difficile diarrhea, irritable bowel syndrome, allergy (can be elaborated here), prevention of urogenital tract infections, inflammatory bowel disease, and candidal vaginitis. Bafeta et al. gathered data from several published trials to examine how harms-related information is reported in publications of randomized controlled trials (RCTs, n=384) of probiotics, prebiotics, and synbiotics. They concluded that harms reporting in published reports of RCTs assessing probiotics, prebiotics, and synbiotics are often lacking or inadequate, and it cannot be broadly concluded that these interventions are safe without reporting safety data [15]. In an epidemiological study of Lactobacillus bacteremia in Finland, the researchers did not find any correlation between the increased probiotic use of L. rhamnosus GG (ATCC 53103) and the incidence of Lactobacillus bacteremia during 1990-2000 [2]. The FDA suggested immunosuppression, structural heart disease, inpatient, pregnancy, and potential for translocation of probiotic across the bowel wall to be potentially at risk for adverse events in probiotic clinical trials.

## Conclusions

Mostly deemed as low-virulence or concomitant, Lactobacillus spp. might be an opportunistic pathogen in immunocompromised, transplant patients and might be related to increased mortality. 
